# Coagulation disorders and the role of tissue factor and tissue factor pathway inhibitor in critically ill patients

**DOI:** 10.1186/2197-425X-3-S1-A297

**Published:** 2015-10-01

**Authors:** F Frantzeskaki, E Merkouri, A Gialeraki, E Paramythiotou, M Theodorakopoulou, O Travlou, A Armaganidis, I Dimopoulou

**Affiliations:** Intensive Care Unit, Attikon University Hospital, Athens, Greece; Psychiko Clinic, Haemostasis and Anticoagulation Laboratory, Athens Medical Center, Athens, Greece; Haematologic Laboratory, Attikon University Hospital, Athens, Greece

## Introduction

Tissue factor (TF), plays a key role in pathophysiology of disseminated intravascular coagulation (DIC) and multiple organ dysfunction syndrome. Tissue factor pathway inhibitor (TFPI) regulates tissue factor activity. Several studies in septic patients have shown that TF production exceeds TFPI production,promoting a prothrombotic state.

## Objective

The aim of our study was to record coagulation disorders, during the course of different stages of sepsis. We also measured TF and TFPI levels, while we investigated the possible correlation of these parameters with morbidity and mortality.

## Methods

This prospective observational study was conducted in a 19-bed polyvalent adult intensive care unit of the “Attikon” University Hospital, between November 2012 and June 2013.TF and TFPA levels were recorded on day of study entry (protocol day 0) and further followed once a week (days 7 and 14 or until they were discharged from ICU or until death). Tissue factor activity (TFa) was measured by homemade test of Stago Diagnostica. TFPI was measured by immunoenzyme assay ELISA standard phase. Disease severity at study entry according to Acute Physiology and Chronic Health Evaluation (APACHE II) score, the degree of organ dysfunction quantified by the Sequential Organ Failure Assessment (SOFA) score, the presence of DIC, as expressed by DIC score, the sepsis stage, and the respective laboratory data, were recorded on each protocol day.

## Results

Twenty nine (19) mechanically ventilated septic patients were enrolled in the study. Among them, 12 patients survived (63%) during ICU staying. Survivors were significantly younger than non-survivors (p = 0.03), with higher disease severity as expressed by APACHE II (p = 0.008). DIC score was significantly higher in non survivors (p = 0.01). Mortality was higher in patients with severe sepsis/septic shock, as compared to patients with sepsis/SIRS (p = 0.02). The latter group of patients showed significantly lower DIC and SOFA scores. TFa has higher in non survivors (p = 0.007). It also showed a trend to be higher in patients with severe sepsis/septic shock, while the difference became significant on study day 14. TFPI tended to be higher in the same group of patients. A significant correlation was observed between TFPI and INR values on study day 0 (Pearson coefficient = 0.9, p = 0.001), between DIC score and APACHE II, and DIC score with SOFA.

## Conclusions

The activation of coagulation in sepsis, as expressed by DIC score, is significantly correlated to organ dysfunction and subsequent mortality. The observed imbalance between TF and TFPI might have a prognostic value. Larger studies are needed, in order to confirm these results, and lead to possible therapeutic considerations targeting to sepsis associated DIC and organ dysfunction.
